# A Brief Review: The Use of L-Ascorbic Acid as a Green Reducing Agent of Graphene Oxide

**DOI:** 10.3390/ma15186456

**Published:** 2022-09-17

**Authors:** Mariano Palomba, Gianfranco Carotenuto, Angela Longo

**Affiliations:** Institute for Polymers, Composites, and Biomaterials, National Research Council, SS Napoli/Portici, Piazzale Enrico Fermi 1, 80055 Portici, Italy

**Keywords:** graphene oxide, reduced graphene oxide, L-ascorbic acid, liquid-phase reduction, gel-phase reduction

## Abstract

The reduced form of graphene oxide (r-GO) represents a versatile precursor to obtain graphene derivatives. Graphene oxide (GO) consists of a layered material based on a carbon skeleton functionalized by different oxygen-containing groups, while r-GO is obtained by the almost complete removal of these oxygen-containing functional groups. The r-GO has mechanical, electrical, and optical properties quite similar to graphene, thus, it proves to be a convenient 2D material useful for many technological applications. Nowadays, the most important aspects to consider in producing r-GO are: (i) the possibility of obtaining the highest reduction grade; (ii) the possibility of improving the dispersion stability of the resulting graphene using surfactants; (iii) the use of environmentally friendly and inexpensive reducing agents. Consequently, the availability of effective soft-chemistry approaches based on a green reducing agent for converting GO to r-GO are strongly needed. Among the green reductants, the most suitable is L-ascorbic acid (L-aa). Different studies have revealed that L-aa can achieve C/O ratio and conductivity values comparable to those obtained by hydrazine, a typical reducing agent. These aspects could promote an effective application strategy, and for this reason, this review summarizes and analyzes, in some detail, the up-to date literature on the reduction of GO by L-aa. The results are organized according to the two most important approaches, which are the reduction in liquid-phase, and the reduction in gel-phase. Reaction mechanisms and different experimental parameters affecting the processes were also compared.

## 1. Introduction

Nowadays, the development of new advanced devices is strictly related to the availability of 2D materials, such as graphene-based materials, because of their unique mechanical, electrical, thermal, and optical properties [1,2,3,4,5,6,7,8,9,10]. Therefore, there is a strong need to develop new schemes for a massive synthesis of these materials, characterized by low-cost and sustainability [11]. Consequently, there is a growing interest in graphene oxide (GO) and its reduced form (r-GO), which is an inexpensive, versatile, printable, and biocompatible precursor of graphene-like materials [12]. GO consists of a layered material based on a carbon skeleton functionalized by different oxygen-containing groups (typically the C/O atomic ratio is less than 3) [13], having physical and chemical properties depending on different parameters, such as the type of oxygen-containing groups, the oxidation level, and the type of graphite used as precursor [14]. The most acceptable structural model proposed for GO is the Lerf–Klinowski model in which the basal planes of GO are decorated by hydroxyl and epoxide groups, whereas the edges are mainly occupied by carboxyl and carbonyl groups in a random manner [14,15,16]. The reduced graphene oxide (r-GO) obtained from the almost complete removal of the oxygen functional groups has mechanical, electrical, and optical properties quite similar to graphene, thus, resulting in a convenient 2D material useful for many technological applications [17,18,19,20,21,22,23,24,25,26].

According to the chemical structures of these layered materials, it is understood that interactions between GO or r-GO sheets and the environment (i.e., molecules, solvents, substrates, embedding polymers, etc.) are strictly related to the presence of oxygen-containing groups and their interaction (i.e., Van der Waals and electrostatic forces). Clearly, GO is predominantly hydrophilic while r-GO is hydrophobic, and the electrostatic interactions are stronger for GO, whereas the Van der Waals interactions play a major role in the case of r-GO because of the increase in unfunctionalized regions [27]. Moreover, the π–π stacking is another important interaction type among the sheets. In fact, each carbon atom of the basal plane bonds with three adjacent carbon atoms with *sp*^2^ hybridized orbitals forming robust *σ* bonds, and the remaining electrons in the *p* atomic orbitals are delocalized all over the basal plane of the sheet forming a strong π bond, which makes possible the π–π stacking interaction [28].

Generally, the conversion from GO to r-GO requires some reducing agents [29,30,31,32], thermal treatment [33,34,35,36], laser-radiation [37,38,39,40,41,42,43,44,45,46], or bacterial methods [47,48,49]. Nowadays, different aspects are taken into consideration when selecting the best way to develop a massive and low-cost production of r-GO. Among the most important aspects, we can include the following: (i) the prospect of obtaining the highest reduction grade, (ii) the possibility of improving the dispersion stability of the resulting graphene using surfactants, (iii) the use of environmentally friendly and inexpensive reducing agents and solvents. So far, it has been proven that commonly used chemical reductants, such as hydrazine or hydrazine hydrate, are highly toxic and explosive, which can potentially induce environmental and safety risks [31,32,33,50]. Consequently, the availability of effective soft-chemistry approaches using a green reducing agent for converting GO to r-GO are strongly needed. Recently, green reductants, such as L-ascorbic acid (L-aa) [51,52,53,54], L-cysteine [55,56,57], glycine [58,59], green tea [60,61,62], various plant extracts [63,64,65], etc., have been studied as reductants for GO. Different studies have revealed that among all cited green chemical reductants, L-aa appears to be the outstanding candidate for achieving a C/O ratio and conductivity values that are comparable to those produced by hydrazine [54], having a mild activity and non-toxic properties. L-aa has proven to have a broad range of applications not only in bio-medical devices but also in electronic devices [51,52,53,54].

In summary, the advantages obtained by using L-aa as a reductant are the following:

- an environmentally friendly and non-toxic reductant;
- a highly efficient removal of the oxygen-groups;
- a low risk of introducing heteroatoms in the reaction products, because L-aa is composed only of carbon, oxygen, and hydrogen atoms.

In addition, preserving the environmentally friendly condition, the reduction process can be carried out in water, the most common and eco-friendly solvent [51]. However, this approach has the disadvantage of the irreversible formation of graphene agglomerates that are not useful for technological applications, and it is difficult to separate r-GO from the solvent and the by-products. Nevertheless, it is well known that a large amount of L-aa can stabilize the r-GO, thus, avoiding the addition of polymeric surface stabilizers and/or surfactants [66].

Recently, an innovative gel-phase technique has been developed to reduce a thin film of GO deposited on a substrate using the L-aa as reductant with the important advantages of avoiding the use of surfactants and being easily available for technological applications [67,68,69,70]. This approach overcomes the limitation of thermal reduction in the presence of a substrate. This limitation is represented by the operating temperature to be applied, which is dependent on the thermal stability of the substrate.

This review analyzes recent studies available in the literature concerning the reduction of GO by the use of an efficient and inexpensive green reductant (L-aa) under different experimental conditions. In addition, some possible mechanisms for the reduction of GO were also discussed. This review could be a useful reference for those scholars involved in this research area proposing its application as the most convenient new green method to reduce GO.

## 2. Reaction Conditions of Reduction of Graphene Oxide by L-aa

This section presents a concise description of the most important experimental results obtained for the reduction of GO by L-aa, their interpretation, as well as the possible filed application of the obtained material. To describe the evolution of this study clearly, the experimental results are presented chronologically and gathered according to the technique used for reduction.

### 2.1. Liquid-Phase Reduction

Zhang et al. (2009) first verified the possibility of developing the reduction of GO via L-aa [51]. The method developed was performed in an aqueous solution at room temperature under vigorous stirring. The study showed the monitoring of the reduction progress by optical absorption spectroscopy. The UV–vis spectrum of GO is typically characterized by the typical π–π* transition peak at 233 nm from the C=C bond, and the *n*–π* transition peak at around 300 nm from the C=O bond [34]. The red-shift of the π–π* transition of GO originates related to the extension of its π-conjugated structure in the r-GO and the decreasing intensity of peaks centered at 300 nm, which is caused by the decrease in the C=O bond, allowing analysis of the reduction. The reduction that occurred was confirmed by FT-IR, Raman, and AFM results. The obtained r-GO, which showed a strongly restacked sheet arrangement with a wrinkled texture, provided a low specific surface area of 11.8 m^2^/g and specific capacity of 128 F/g at a current density of 50 mA/g [51].

In the same period, Gao et al. (2010) presented a “green” reduction of GO using L-aa, analyzing the possible use of L-tryptophan as a stabilizer, to produce a stable dispersion of r-GO in an aqueous solution [66]. In brief, an aqueous solution of GO, L-aa, L-tryptophan, and NaOH was treated by ultrasonication at 80 °C for 24 h. After that, the mixture was cooled to room temperature, followed by another 1 h of ultrasonication. Thus, a large amount of stably dispersed aqueous r-GO was easily obtained. The experimental results showed the efficiency of L-tryptophan as a stabilizer to avoid the agglomeration and precipitation of the resulting r-GO sheets. L-tryptophan contains an electron-rich aromatic group that can function as an electron donor and be absorbed onto the r-GO sheet, based on the π–π interaction. In the meantime, the terminal carboxylic acid can supply enough negative charge, and the electrostatic repulsion can make the r-GO dispersions stable [66].

Successively, Fernández-Merino et al. (2010) [54] verified the use of L-aa to obtain stable suspensions of highly reduced r-GO in some common organic solvents, such as N,N-dimethylformamide (DMF) and N-methyl-2-pyrrolydone (NMP). In addition, the comparison of the deoxygenation efficiency of GO by different reductants (i.e., hydrazine, sodium borohydride, and pyrogallol) showed that only L-aa was found to yield highly reduced suspensions in a way that was comparable to those provided by hydrazine [54].

Sui et al. (2011) analyzed the effect of the amount of L-aa to reduced GO hydrogel and demonstrated that the mass ratio of L-aa to GO, temperature, and pH value of the reaction mixtures play a significant role in the formation of irreversible r-GO agglomerates in the form of hydrogels [71]. The hydrogels consist of a 3D cross-linked network of r-GO sheets self-assembling into a well-defined and interconnected 3D porous network through π–π interaction during gelation. Furthermore, this study demonstrated that r-GO in the form of a hydrogel with an excess of L-aa as a bioactive component can easily be used to release it in a diffusion-controlled manner. After the complete release of L-aa, the hydrogel exhibits excellent mechanical and electrical properties, and for this reason, it can be advantageously used in the fields of tissue engineering, drug delivery, soft machines, regenerative medicine, biosensors, etc. [71].

A complete study about the pH effect on the morphology of the as-prepared hydrogel r-GO was presented by Ha et al. (2019) [72]. This work shows that the reduction of GO in the liquid phase was completed in 1 h. The morphological characterization performed by scanning electron microscopy (SEM) demonstrated the formation of the 3D cross-linked spherical structure of r-GO by the hydrogel process with a diameter from 4 to 2 μm and with a specific surface area of 150 m^2^/g, when the pH of the solution was 2. The experimental measurement showed that more spherical and compact structures were obtained at pH 10 with an improved specific surface area of 216 m^2^/g.

The effect of pH on the degree of dispersion, packed with r-GO, can be explained by zeta potential analysis. The colloid of GO dispersion has a zeta potential −43 mV when the pH is 10 [73]. It is well kwon that Zeta potential values greater than −30 mV are generally considered to exhibit sufficient mutual repulsion to ensure the stability of dispersion [74]. When the droplets are generated from the GO colloid fabricated at pH 10, a more densely compacted structure of r-GO can be formed by the capillary-force-driven self-assembly of well dispersed GO sheets in the droplet during the solvent evaporation. Similar experimental results for the formation of the r-GO hydrogels were confirmed by Kondratowicz et al. (2017) [75]. This study explained that the by-products of reduction, such as dehydroascorbic acid and water molecules, may form additional hydrogen bonds with residual carboxylic groups on r-GO planes and contribute to the final structure of hydrogels [75].

Successively, the influence of pH and surfactants used for reduction by L-aa on the adsorption mechanism of organic contaminants, such as phenanthrene (a representative nonpolar, nonionic, and aromatic contaminant) and 1-naphthol (a representative polar, aromatic contaminant) was studied by Wang et al. in 2019 [76]. The study shows that the pH of the solution had a negligible effect on phenanthrene adsorption for both GO and r-GO, and it inhibited 1-naphthol adsorption at high pH because of the electronic repulsion. This was mainly attributed to the hydrophobic interaction, π–π interaction, and H-bonding between graphene sheets and organic contaminants. The same study shows that the use of the surfactants had different effects on the adsorption of polar and nonpolar aromatics onto graphene materials. For sodium dodecyl benzene sulfonate (SDBS), the exfoliation effect could enhance the adsorption affinity; thus, it could counteract the inhibition effect caused by competition. Cetyltrimethylammonium bromide (CTAB) could form hemimicelles on reduced graphene oxide, which may provide a favorable media for organic contaminant partitioning. In addition, this study shows that the r-GO could be regenerated and reused with high recyclability over five cycles. These findings could provide a promising material for wastewater treatment and the understanding of the fate and transport of organic contaminants in aquatic environments.

De Silva et al. (2018) [77] monitored the reduction of GO by L-aa in an aqueous solution in 10-min intervals up to 1 h in order to study how structural and morphological changes would take place. The reduced products obtained at different time periods were characterized in detail by UV–visible spectroscopy, X-ray diffraction (XRD), X-ray photoelectron spectroscopy (XPS), attenuated total reflectance Fourier transform infrared (ATR-FT-IR) spectroscopy, Raman spectroscopy, thermogravimetric analysis (TGA), atomic force microscopy (AFM), and scanning electron microscopy (SEM). The UV–visible spectra displayed a complete removal of the GO peak by 50 min, while other characterization techniques revealed the presence of residual oxygen functionalities. In particular, the XPS results showed that the decline in the oxygen atomic percentage was mainly due to the removal of the hydroxyl and epoxy groups located at the basal planes of the GO sheets and, to a small extent, due to edge carbonyl groups. AFM characterization indicated that at the intermediate stages of reduction, both GO and r-GO coexist in the material, as confirmed by XRD results.

The study of the impact of ultrasounds on the rate of GO reduction in L-aa aqueous solutions was carried out by Abulizi et al., 2014 [78]. They found that the r-GO formation under ultrasound treatment was accelerated in comparison with the conventional mechanical mixing treatment. To understand the effects of ultrasound, the authors compared the experimental results on the trend rates of r-GO formation, as a function of temperature, under ultrasound and mixing treatment, showing that this rate was increased by ultrasound treatment. The authors proposed that physical effects such as shear forces, microjets, and shock waves during acoustic cavitation enhanced the mass transfer and reaction of L-aa with GO to form r-GO, as well as the change in the surface morphology of GO. Furthermore, the rates of r-GO formation were suggested to be affected by local high temperatures of cavitation bubbles [78].

Similarly, the increase in kinetics and in the GO reduction degree under UV irradiation were analyzed by Go et al., 2018 [79]. They demonstrated that when the reactant solution was placed under UV irradiation (254 nm), L-aa improved its chemical activity caused by its UV-sensitive oxidation property [80]. In particular, the reduction was performed under various conditions, (i) without any reducing agent, (ii) using L-aa, (iii) using L-aa under UV irradiation (254 nm), and monitored by using UV–visible spectroscopy up to 24 h to explore the effect of UV irradiation on the reduction of GO by L-aa. The evolution of UV–Visible spectra for these different conditions, confirmed that the UV irradiation (254 nm) improves the activity of L-aa.

In fact, the UV–visible spectrum of GO showed the typical π–π* transition peak at 233 nm from the C=C bond, and the *n*–π* transition peak at around 300 nm from the C=O bond [33]. The red-shift of the GO π–π* transition can be used as an indicator of its reduction [51]. Consequently, analyzing the trend of this shift for all three different reduction conditions, it is possible to deduce the advantageous effect of UV radiation on a reduction of GO [79,80].

To further understand the commonality of the procedures developed and presented in this review, Table 1 lists the main information about the parameters used in all reported studies. In particular, Table 1 lists the solvent used, the weight ratio between GO and L-aa, the pH value of solution, the presence of a stabilizer, the temperature and time of reaction, and the main experimental characterizations.

In many cases, the approach to reduce GO in an aqueous solution by L-aa was used to prepare functional and advantageous nanocomposite materials based on r-GO for many application fields. A few examples are shown below.

Ding et al. in 2015 [81] used L-aa to realize r-GO–TiO_2_ composite films as a photoanode in DSSC. The experimental results demonstrated a 30% increase in conversion efficiency compared to that of the pure TiO_2_ photoanode [81].

Dan et al. in 2018 [82] examined three types of r-GO/polyhydroxy butyrate (PHB) composites by employing three reducing agents: sodium borohydride, hydrazine, and L-ascorbic acid. The electrical properties of the r-GO/PHB composites achieved by L-aa were comparable to the best values known in the bio-composite field.

As far as we know, studies associated with the utilization of r-GO in osteogenesis indices by electrical stimulation have rarely been reported. Xiong et al. in 2017 [83] presented the study on the preparation of reduced graphene oxide/zinc silicate/calcium silicate (r-GO/ZS/CS) by L-aa with an optimal surface electroconductivity. The conductive biocomposite obtained was analyzed in vitro osteogenesis of mouse bone mesenchymal stem cells.

### 2.2. Gel-Phase Reduction

Recently, a gel-phase technique has achieved a growing interest. This technique is based on the reduction of a thin film of GO deposited on a substrate by the diffusion of L-aa molecules in it. The peculiarity of this approach is that GO is not dispersed in a solvent, but it is swollen by water, and it persists in the form of coating stacked to the substrate, and the chemical interaction with L-aa takes place by permeation of the reductant in these lamellar structures. This approach represents an important technological breakthrough because the development of many functional devices can be made directly by the reduction of a large area of GO coating deposited on selected substrates (i.e., polymers, glass, etc.) [52,68,69,70,84]. In addition, considering that this approach allows the GO reduction by L-aa at a low temperature, it is possible to overcome the temperature limitation due to the thermal stability of the substrate. This method preserves the properties of substrates, and it avoids the use of surfactants.

**Figure 1:**
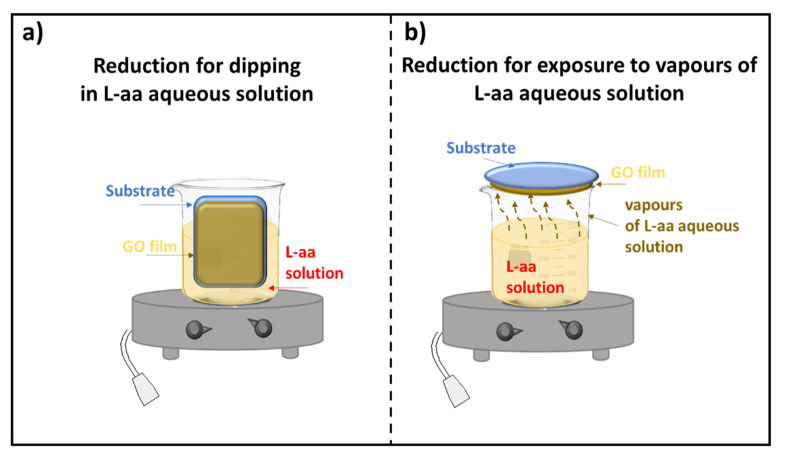
Scheme of the reduction of a GO film deposited on a substrate (**a**) by dipping; (**b**) by exposure to vapors of a L-aa solution.

According to the literature, only a few methods have been developed for the reduction of GO thin films. Two approaches for reducing GO film deposited on a substrate were described: the first one is based on the dipping of supported GO in a reducing L-aa solution, and the second method requires the exposure of supported GO to vapors of a reducing L-aa solution. Both procedures, which could need a controlled temperature, are shown in Figure 1.

Liu et al. in 2015 [52] first reported the use of L-ascorbic acid/water vapor as a reducing agent for GO films. In this paper, GO on cellulose was placed on the top of a glass bottle in a Teflon-lined autoclave containing different concentrations of an aqueous solution of L-aa. Finally, the autoclave was heated at 100 °C for 48 h. The same procedure was used to prepare r-GO–Ag composites that can be used for active substrate surface-enhanced Raman scattering and as antibacterial material.

In 2016, Li et al. [70] published one article concerning the preparation of porous r-GO membranes reducing GO on copper hydroxide nano-strand freestanding membranes by dipping in a 60 mL L-aa aqueous solution heated at 90 °C for 4 h. The results confirmed that a porous r-GO membrane, fabricated from a graphene oxide sheet via etching copper hydroxide nano-strands by L-aa reduction, provides an effective structural configuration for enhancing its gauge factor.

Tas et al. in 2019 [69] proposed the reduction of graphene oxide thin films deposited on glass by dipping in an opportune solution of the L-aa at low-temperature. To compare the effectiveness of the reduction process, hydrazine hydrate was also used as a chemical reducing agent following the same method. The results have shown that this reduction process, which does not contain heavy toxic chemicals and does not require nitrogen, argon, etc., is more successful.

Chen et al. in 2020 [84] reported on the preparation of cellulose/r-GO aerogels for the development of chemical vapor sensors. For the GO reduction, the cellulose/GO hydrogels were put in an aqueous solution of L-aa at 95 °C for 2 h. Sensors based on these aerogels exhibited fast response, good recovery, high sensitivity, and excellent reproducibility. The inexpensive, easy, green, and scalable preparation of this new type of vapor sensor could be expected to lead to new sensing and biomedical applications.

**Figure 2:**
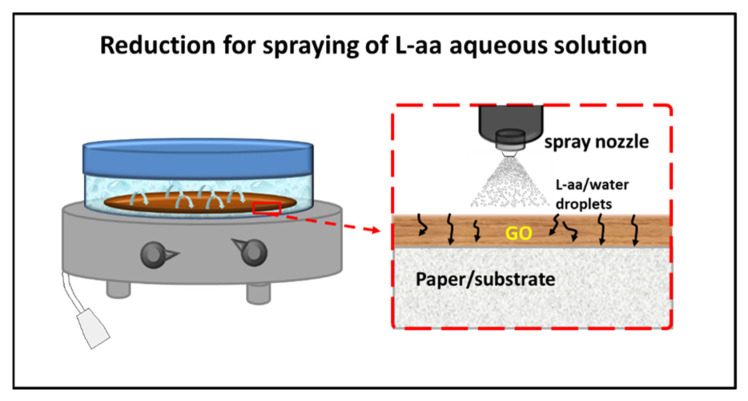
Scheme of the reduction of GO film deposited on paper/substrate by spraying of a L-aa aqueous solution [67].

In the same year, Longo et al. [67,68] published a new method for a green gel-state chemical reduction of GO supported on cellulose substrates. The possibility of having an effective mass transport of the reductant inside the swollen GO deposit was ensured by spraying a reducing solution of L-aa on the GO film, allowing it to reflux for 48 h in a closed microenvironment at 50 °C. A scheme of the apparatus used for reduction is shown in Figure 2.

The principal information on the parameters selected in the above-described gel-phase approach are summarized in Table 2. In particular, the method used to reduce the GO on the substrate, the concentration of the L-aa in water, the selected substrate, the temperature and time of reaction, and the characterization techniques are given.

#### The Reduction of GO Film Deposited on Paper/Substrate Spraying of L-aa Aqueous Solution

This gel-state reduction technique, based on spraying an L-aa aqueous solution, represents a convenient approach for a complete reduction of the GO layers supported on thermally unstable substrates [67,68]. The most important experimental results published in previous manuscripts [67,68] can be summarized as follows. In addition, in order to improve the previous results, a quantitative analysis of the degree of oxidation of GO before and after the reduction is presented.

According to the thermogravimetric investigation, the process temperature selected (i.e., 50 °C) is necessary to increase the mobility of the water and L-aa molecules in the GO inter-layers [68]. The SEM investigation confirmed a structural modification of the GO coating after the treatment, mainly consisting of an increase in the coating flatness. In addition, SEM confirmed a strong interfacial adhesion between the GO/r-GO coating and the fibrous substrate. This micro-structural characteristic, due to an excellent adhesion at the GO–paper interface, is relevant for achieving a highly flexible r-GO layer supported on paper, and it is vitally necessary for industrial exploitation [68].

The XRD results of r-GO/paper show the presence of the main peaks of the r-GO pattern combined with the XRD signals of residual GO. In particular, the results showed that the obtained r-GO coating is composed of platelets with an average thickness of ca. 27 nm and a width of ca. 40 nm, which are aligned parallel to the interfacial plane and show good graphitic quality [67]. These experimental results confirmed the obtained reduction of GO on paper only qualitatively.

To establish the degree of oxidation of GO before and after the reduction, the Fourier transform infra-red spectroscopy was recorded in attenuated total reflectance (ATR) mode in the 4000–700 cm^−1^ range by using a spectrophotometer (PerkinElmer Frontier NIR, Milan, Italy).

**Figure 3:**
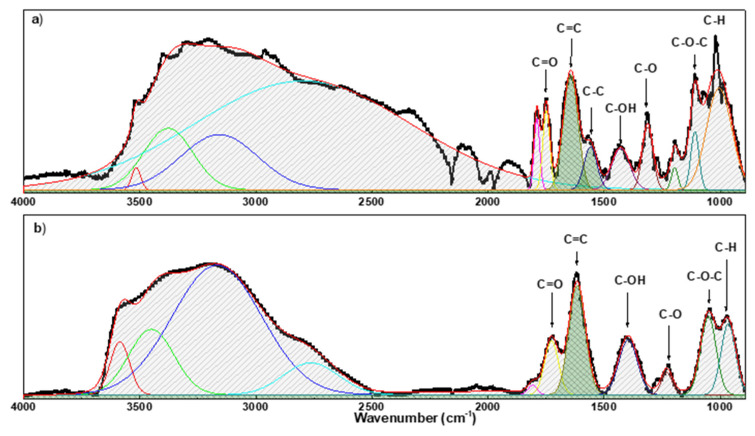
FT-IR spectrum of GO on paper/substrate (**a**) and r-GO on paper/substrate (**b**).

The ATR spectra (see Figure 3) showed that most of the peaks referring to oxygen-containing groups are present in the GO layer. In addition, the broad peak in the spectra in the region 3740–3100 cm^−1^and the peak at ≈1640 cm^−1^ can be referring to the water molecules absorbed on the interlayers of GO. Table 3 details the main peaks measured by spectra.

To perform a quantitative analysis of the degree of oxidation of GO before and after the reduction by ATR spectra, the following procedure proposed by Guerrero-Contreras et al. [85,86] was used:

- A polynomial baseline was calculated and subtracted from the raw spectra.
- The resulting spectra were multiplied by −1 in order to have positive bands.
- The peak deconvolution was obtained by Gaussian fit to achieve the peak area.

The comparison between the spectrum before and after reduction shows a general decrease in intensity of the peaks related to oxygen functional groups. At this point, the degree of oxidation of GO was evaluated by calculating the relative percentage of oxygen-containing functional groups (*RPox*) compared to the presence of all functional groups observed in the wavenumber range of 900−1850 cm^−1^ (for all peaks in Figure 3 [85,86]). *RPox* was calculated using the following formula:

(1) $$RPox=\left[\frac{sum\;of\;area\;of\;all\;peaks-area\;od\;C=C\;peak}{sum\;of\;area\;of\;all\;peaks}\right]*100$$

The analysis reveals that the *RPox* decreases from 68% to 37% after the reduction.

## 3. Reduction of GO by L-aa

Nowadays, the mechanism of the chemical reaction between GO and L-aa is not completely understood. This section provides a detailed description of the mechanism given in the literature for the GO reduction by L-aa and describes the effect of UV light on this mechanism.

### 3.1. Possible Mechanism of Reduction

**Figure 4:**
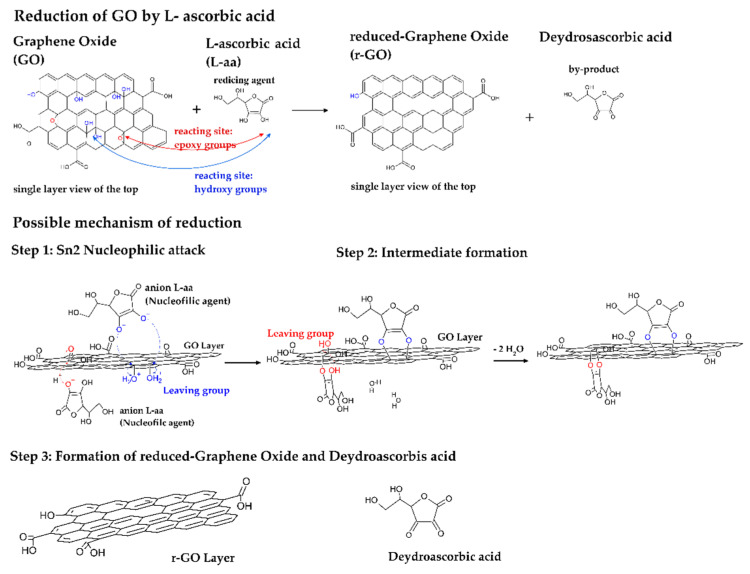
Schematic representation of the reaction pathway [67].

According to the literature [29,66], two different reactions are involved in the reduction of GO steps. The first reaction involves the reduction of GO vicinal-hydroxyls by L-a. The second one involves the reduction of epoxy groups by L-aa. Both reaction pathways are shown in Figure 4. In particular, the electron density withdrawing from the five-membered ring of L-aa makes the hydroxyls contained in this molecule much more acidic, consequently the L-aa can dissociate, providing two protons, which are transferred to GO, while nucleophilic species (i.e., the oxyanion of L-aa: C_6_H_7_O_6_^−^) are generated. Figure 4 shows that both reacting sites require the formation of a good leaving group, which is a hydroxyl group in the case of epoxies and a water molecule in the case of vicinal-hydroxyl groups. These types of leaving group are generated by protonation of the cited GO reactive groups (i.e., vicinal hydroxyls and epoxy groups). After this preliminary acid–base reaction, a SN2 reaction step follows. In this reaction, the nucleophilic agent attacks the *Sp*^2^-carbon of the epoxy group or the α-*Sp*^3^-carbon of the alcoholic groups, and hydroxyl or water results as a by-product, respectively. In the case of the reaction involving epoxy, a further condensation by the SN2 mechanism follows. Then, this intermediate undergoes a thermally induced red-ox reaction, which leads to the formation of: reduced graphene oxide (r-GO), and as by-products, dehydroascorbic acid and water molecules [29,66]. Some studies report that dehydroascorbic acid (C_6_H_6_O_6_) can be further converted to guluronic and oxalic acids [59,86], and then CO_2_, CO, and water are generated during GO reduction to r-GO.

**Figure 5:**
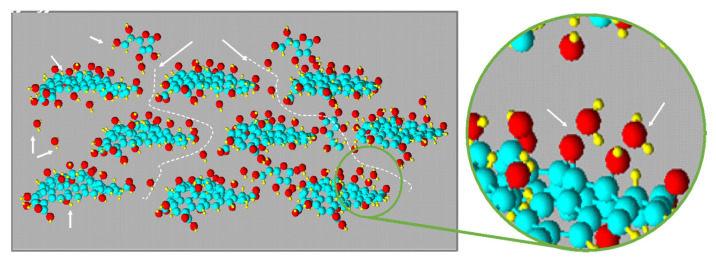
Representation of swollen GO and the diffusion pathway of a L-aa aqueous solution.

A similar chemical mechanism, proposed by Longo et al., 2020 [67], took place when a L-aa aqueous solution is sprayed on a GO thin film. The presence of a large amount of oxygenated functional groups on GO makes the material very hydrophilic and, thus, provides it with the capability to absorb water and swell [87,88]. In fact, the absorption of water molecules on the GO layer by physical interaction with the epoxide and/or hydroxyl groups is a well-known phenomenon (see Figure 5). This absorption gives the layered stacked GO a natural tendency to swell as a consequence of the enlarged inter-layer spacing. At room temperature, a slow diffusion rate of L-aa molecules, which always characterizes a gel-phase reduction, and consequently, a lower value of the reaction rate, is expected. To increase both the mobility of water and the corresponding mobility of L-aa molecules in the GO channels, the process temperature was increased to 50 °C.

### 3.2. Possible Influence of UV-Irradiation

This eco-friendly approach can be improved by using L-aa as a photosensitive reducing agent. Go et al. demonstrated that when L-aa was excited by UV irradiation at 254 nm, it oxidized with simultaneous deprotonation and this proton-coupled electron transfer was capable of inducing a chemical reduction of GO [79,88]. Therefore, UV irradiation of L-aa is expected to accelerate the reduction of GO. This method can be advantageously used for developing eco-friendly and scalable processes [79,88].

## 4. Discussion, Conclusions, and Future Perspectives

**Figure 6:**
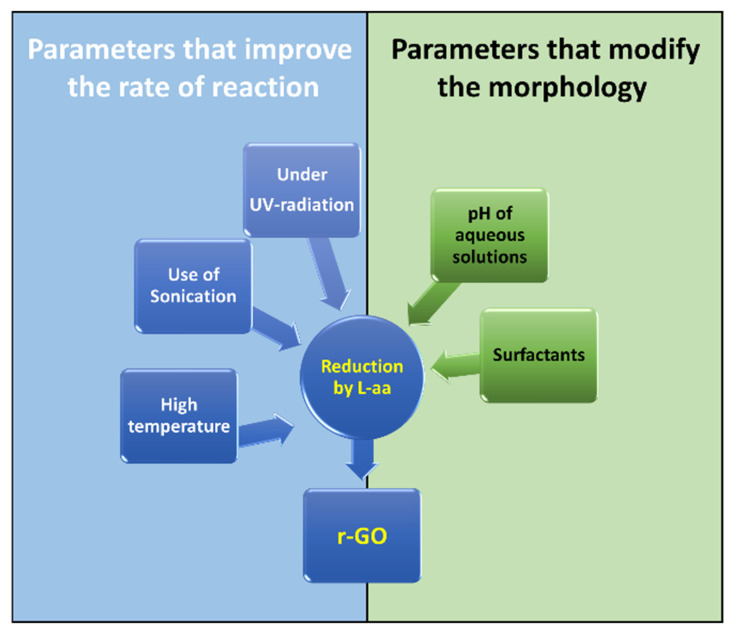
Parameters that act on the reduction mechanism.

The aim of this review is to highlight the potential for using L-aa as a green reducing agent to improve eco-friendly and large-scale production of r-GO. Liquid-phase and gel-phase reductions were briefly discussed here. As far as the first approach is concerned, experimental results have demonstrated the advantageous use of some factors in improving the reduction process shown in Figure 6. Higher temperature, the use of sonication, and exposure under UV radiation are factors that (separately or simultaneously) allow an increase in reduction. Furthermore, this review presents an analysis of the role of the pH of the reaction mixture on the formation of irreversible r-GO agglomerates and the use of surfactants to modify the adsorption properties of r-GO. Many studies have demonstrated that this approach can be advantageously used to obtained functional nanomaterials based on r-GO

More recently, there has been a growing interest in the potential for developing reduction processes in the gel-phase. This approach overcomes some limitations of the reduction of GO in the liquid-phase, such as the isolation of r-GO from the solution. In addition, this approach can be used to reduce a large area of the GO coating deposited on selected substrates (i.e., polymers, glasses, etc.), and for this reason, the approach may provide many technological breakthroughs.

Table 4 compares the two approaches by outlining and summarizing the pros and cons for both.

Finally, the review shows the efficiency of L-aa as a reductant agent. In the future, this green reduction method of GO may provide fascinating results in terms of graphene quality, size, and also production.

The aim of this review was to collect all the literature papers concerning the most important approach for the GO reduction based on the use of L-aa. The achieved r-GO quality allows for the technological exploitation of this nanostructure in a variety of forms (coating, self-supported, embedded in a polymer, etc.). These factional materials have potential applications for flexible electronics, sensing applications, optics applications, etc. In particular, the use of this green chemical method is an emerging technology of a fundamental importance for the production of large-area, lightweight, low cost, and mechanically stable devices. The knowledge of all aspects related to the synthesis and properties of r-GO obtained by the L-aa reduction technique is a critical point for bringing this process to mass production.

## Figures and Tables

**Table 1:** Summary of the principal parameters used in the cited methods of the liquid-phase reduction of GO.

Solvent	GO ÷ L-aa	Base for pH	Stabilizer	Temperature and Time	Characterizations	Reference
water	0.05 ÷ 50	—	—	23 °C for 48 h under stirring	UV–Visible, AFM, TEM, Raman, FT-IR	Zhang et al., 2009 [51]
water	0.2 > 10	pH 9–10 with NaOH	L-tryptophan	80 °C for 24 h under sonication for 0.5 h	AFM, XPS, Raman	Gao et al., 2010 [66]
water, NMP, DMF	—	in case of water pH 9–10 with 25% NH_4_OH	—	95 °C for 15, 30, 180, 240 min	ATR-FTIR, XPS, TGA, UV–Vis	Fernández-Merino et al. [54]
water	From 1:832 To 8:1	pH 7–8 with several drops of 0.1 M HCl solution	—	Without stirring, for a few hours, from 25 °C to 80 °C	XPS, SEM	Sui et al. [71]
water	1.2	pH 2–10 was adjusted using NH_3_·H_2_O		various reaction times of 5, 15, 30, and 60 min, T 95 °C	SEM, UV–Vis	Ha et al. [72]
water	—	—	SDBS, CTAB	stirred at 500 rpm for 72 h	XPS, FT-IR, Raman	Wang et al. [76]
water	—	pH to 10 with NH_3_·H_2_O	—	Under stirring, 65 °C, 1 h	XRD, SEM, XPS	De Silva et al., 2018 [77]
water	—	pH to 11 with NaOH	—	High-power horn-type ultrasound sonicator	UV–Vis, FT-IR, SEM	Abulizi et al., 2014 [78]
water	—	pH to 10 with NH_3_·H_2_O	—	UV light (254 nm) was irradiated at 2 mW/cm^2^ power for 24 h	UV–Vis, FT-IR, Raman, XPS	Go et al., 2018 [79]

**Table 2:** Summary of principal parameters used in the cited methods of the gel-phase reduction of GO.

Methods	Concentration of L-aa	Substrate	Temperature and Time	Characterizations	Reference
Exposure to vapors	Different concentrations	Cellulose	100 °C for 48 h	UV–Visible, FT-IR, Raman, AFM, XPS	Lui et al., 2015 [52]
Dipping	30 mg/mL of L-aa in water	Freestanding membrane	90 °C for 4 h	UV–Vis	Li et al., 2016 [70]
Dipping	Different concentrations	Glass	95 °C for 15 min	XRD, Raman, XPS	Tas et al. [69]
Dipping	30 mg/mL of L-aa in water	Paper	95 °C for 2 h	XRD, Raman, XPS	Chen et al. [84]
Spraying		Paper	50 °C for 48 h	XRD, FT-IR, SEM	Longo et al., 2020 [67] Palomba et al., 2021 [68]

**Table 3:** ATR peaks assignments for GO and r-GO on paper.

Vibration Mode	GO	r-GO
	Position	Position
O-H stretching, water molecules	3740–3100	3740-3100
C-H stretching	2800–2600	2766
C=O stretching carbonyl functional groups located on the edge of the sheets (yet COOH and C=O);	1753 1647	1726 1620
C=C aromatic skeletal stretching		
CH_2_ deformation stretching	1433	1415
C-OH stretching bending mode of hydroxyl groups	1431	1404
C-OH stretching	1239	1227
C-O-C stretching epoxy groups	1150	1147
C-H bending aromatic skeletal	971	965

**Table 4:** Summary of principal advantages and disadvantages of liquid-phase and gel-phase approaches.

*Liquid-Phase Reduction*		*Gel-Phase Reduction*	
Advantage	Disadvantage	Advantage	Disadvantage
**✓** Uniform degrees of reduction	Separation of r-GO from solvent	**✓** Treatment of a large area	Need to control the diffusion of L-aa to have a uniform degree of reduction in the coating
**✓** Control of morphology	Re-dispersion of r-GO in selected matrix to possible applications	**✓** Preparation of r-GO coating in one-step	
		**✓** Use of thermally unstable substrates	
